# Fault Diagnosis Method for Rotating Machinery Based on Threshold-Free Recurrence Distance Visualization Convolutional Neural Network

**DOI:** 10.3390/s26123815

**Published:** 2026-06-16

**Authors:** Chao Song, Fuzhou Feng, Feng Liu, Ziyu Liu, Hao Hu

**Affiliations:** 1 Department of Vehicle Engineering, Army Academy of Armored Forces, Beijing 100072, China; fengfuzhou@tsinghua.org.cn (F.F.); zgyliufeng@163.com (F.L.); huhao702@163.com (H.H.); 2 Beijing General Institute of Electronic Engineering, Beijing 100143, China; 13662165123@163.com

**Keywords:** fault diagnosis, recursive plot, Threshold-Free Recurrence Distance, CNN

## Abstract

Recursive Plots (RPs) can fully utilize the information of signals on a time scale, but their application involves the issue of manual threshold selection, and different thresholds have a significant impact on the analysis results of recursive plots, which in turn affects the accuracy of subsequent fault diagnosis models. Some scholars have proposed the no-threshold recursive plot method to address the above issues, but this method is not comprehensive enough and has limitations. On the basis of RPs, this article proposes a Threshold-Free Recurrence Distance (TFRD), which is combined with a Convolutional Neural Network (CNN) to form a TFRD-CNN rotating machinery fault diagnosis model. The accuracy of the method is tested using bearing vibration data from Western Reserve University, and the effectiveness of the model is verified using a planetary gearbox gear fault dataset. At the same time, the TFRD-CNN method is compared with a Markov Transition Field (MTF), Gramian Angular Fields (GAF), and RP and URP combined with CNN methods. The results show that the TFRD-CNN method has significant advantages.

## 1. Introduction

As rotating machinery structures grow increasingly complex and integrated, the demands placed on bearings and gears—key rotating components—continue to escalate. Ensuring the proper functioning of bearings and gears is of critical importance in rotating machinery. The ability to swiftly identify and diagnose faults in bearings or gears during the operational phase of rotating machinery serves as a vital assurance for enhancing work and production efficiency while simultaneously reducing production and maintenance costs. At present, the prevalent technology for fault diagnosis in rotating machinery involves the extraction of vibration signal features combined with classification and recognition algorithms. Feature extraction techniques for vibration signals primarily encompass fast Fourier transform, wavelet transform, and empirical mode decomposition, among others. Classification and recognition algorithms mainly include support vector machines, random forests, deep learning algorithms, and similar.

In the realm of deep learning algorithms, the Convolutional Neural Network (CNN) stands as a prominent type of feedforward neural network, extensively employed in image recognition and various visual tasks, and constituting one of the pivotal cores of deep learning [1,2]. It emulates the visual processing mechanism of the human brain, thereby enhancing the accuracy and intelligence of computer-based image data recognition. Presently, CNNs have found widespread applications across diverse domains, including medical image analysis, object detection and localization, facial recognition, security surveillance, agricultural and industrial inspection, smoke detection and fire alarm systems, as well as image compression and reconstruction.

CNN has great advantages in recognizing image data, and it is a commonly used method to convert rotating machinery vibration data into two-dimensional image data and combine it with CNN for fault diagnosis and classification. Aiming at the non-stationary characteristics of diesel engine vibration signals, Li [3] et al. converted vibration signals into two-dimensional images using Gram angle fields, and then used convolutional neural networks for adaptive feature extraction and fault recognition. The average fault diagnosis accuracy reached 98.4%, and the stability of the method was verified by comparing the recognition effects under different signal-to-noise ratios and experimental conditions. Li [4] et al. proposed a fault diagnosis method combining short-time Fourier transform and a convolutional neural network, which takes the time–frequency spectrum obtained by Fourier transformation of bearing vibration signals as samples, and then uses a convolutional neural network for diagnosis, achieving end-to-end fault pattern recognition. Xie [5] et al. also proposed a roller fault diagnosis method that integrates a short-time Fourier transform and a convolutional neural network. The fusion model constructed achieved an accuracy of 99.6% in identifying roller faults, and the reliability of the method was verified through experiments conducted in mines. Xun [6] et al. used Markov transition fields to convert time-series data into image data and proposed a model for bearing fault diagnosis using a two-dimensional wide convolutional neural network. The model was tested using data from Western Reserve University and diagnosed on actual turntable bearings, verifying its generalization performance. Mario [7] et al. compared the fault diagnosis accuracy of images obtained through time–frequency transformation and SDP transformation combined with CNN, and verified through two publicly available bearing datasets that SDP-CNN has superior diagnostic accuracy and computation time. Afterwards, Mario [8] et al. proposed a combination of enhanced SDP-CNN, which first filters the vibration signal, then performs SDP transformation and combines it with optimized CNN for classification; the results showed the applicability of the new pipeline in terms of percentage accuracy and ROC curve compared to the classical approach.

To enhance the substitutability of image data in place of time-domain vibration data, researchers have increasingly adopted recursive plot encoding of time-domain vibration data for fault diagnosis purposes. The recursive plot-based deep neural network methodology involves transforming one-dimensional time signals into two-dimensional recursive images, which are subsequently subjected to classification and recognition via deep learning architectures. Extensive research has been conducted in this domain by numerous scholars. Initially, Liang [9] and colleagues leveraged the capability of recursive plots to analyze non-stationary nonlinear signals in conjunction with the feature extraction strengths of convolutional neural networks (CNNs) to develop a structural damage identification approach under non-stationary excitation conditions. The efficacy and robustness of this methodology were validated through numerical simulations involving simply supported beams. Subsequently, Shang [10] et al. converted one-dimensional vibration signals into recursive plots and employed CNNs to extract and classify fault features in diesel engines. Shi [11] and their team proposed a hybrid model integrating recursive plots with enhanced deep residual networks for bearing fault diagnosis, addressing the susceptibility of bearing vibration data to noise interference. Their model exhibited superior generalization and noise resistance compared to other deep learning approaches. Zhang [12] et al. developed a bearing fault diagnosis framework combining recursive plot encoding with residual networks, demonstrating high diagnostic accuracy. Wang [13] et al. proposed a rotating machinery fault diagnosis model based on a rainbow recursive graph, which converts one-dimensional vibration signals into color images to capture more fault information. Combined with a convolutional neural network based on LeNet-5, the accuracy of fault recognition reached 97.86% on public datasets. Zhang [14] et al. proposed a fault diagnosis method based on a combination of multiple denoising and recursive plots to address the problems of difficult feature extraction and low time information utilization in transformer vibration signals under noise interference. The method uses a fully adaptive noise set empirical mode decomposition combined with adaptive wavelet thresholding to perform multiple denoising on transformer vibration signals, and then constructs the denoised signal into recursive plots. Combined with convolutional neural networks, the method achieves transformer fault diagnosis with a final diagnostic accuracy of 98.125%. Zhao [15] et al. encoded the collected vibration data of oil pipeline flanges into recursive plots, and combined them with an adaptive deep residual network that integrates ECA (efficient channel attention) and efficient channel attention mechanism for fault diagnosis. The results showed that the proposed method has better accuracy, robustness, and computational performance compared to existing methods. Li [16] et al. proposed a rolling bearing fault diagnosis model using multi-scale asymmetric recursive plots combined with a dual-channel-enhanced residual shrinkage network to address the issue of feature redundancy in processing long sequences. The accuracy of the model was verified using the Jiangnan University bearing dataset, with a diagnostic accuracy of 98.75%. Wan [17] et al. first decomposed the vibration signal into intrinsic modal components using empirical mode decomposition, then reconstructed the signal based on the correlation between the modal components and the original vibration signal, encoded and converted it into a relaxed recursive plots, and finally used convolutional neural networks to complete classification and recognition.

The above researchers have gradually transitioned from using only recursive graphs combined with deep learning algorithms to improving recursive graphs. However, there are still some shortcomings in the recursive graphs themselves, especially in manually selecting thresholds, which significantly affects the preservation of feature information and diagnostic accuracy. In order to reduce the impact of threshold selection on diagnostic results, researchers have proposed a Threshold-Free Recurrence Plot method aimed at eliminating diagnostic bias related to threshold selection. Yan [18] et al. combined this method with CNN for pulse signal analysis, achieving a classification accuracy of 98.14%, but did not study vibration signals. Chen [19] et al. proposed a Threshold-Free Recurrence Plot encoding technique and successfully achieved accurate gear fault identification using adaptive normalized CNN. Despite these advances, the universality of these methods remains an area that requires further research.

In summary, as a representative of deep learning methods, Convolutional Neural Networks (CNNs) [20] exhibit outstanding feature self-learning capabilities [21,22,23], with strong adaptability and efficient computational speed. This article focuses on the fault diagnosis of rotating machinery equipment. Based on the advantages of CNNs in image recognition and the basic principles of recursive graphs, a new Threshold-Free Recurrence Distance convolutional neural network (TFRD-NN) method is proposed for rotating machinery fault diagnosis. This method modifies the process of generating recursive graphs by normalizing vibration data, constructing phase space, and using a direct encoding method based on the square difference between two points in the phase space. Therefore, this technique avoids the influence of threshold selection on recursive graphs. We validated the method of using bearing vibration data and gearbox gear peeling fault vibration data through CNN-based feature extraction and classification. The experimental results compared with other methods confirm that this method has high comprehensive fault diagnosis accuracy.

The rest of this article is organized as follows. In Section 2, the basic theory of the method in this paper is given. Section 3 illustrates the efficacy of the proposed method in different datasets. Section 4 concludes this article.

## 2. Construction of Threshold-Free Recurrence Distance Convolution Neural Network Model

Recursive Plots (RPs) were proposed by ECKMANN et al. [24] in 1987. The phase space reconstruction originally used to describe system dynamics can reflect the autocorrelation of signals on a time scale [25]. The core idea is to embed one-dimensional time series data into a higher dimensional phase space, and construct a matrix by comparing the distances between points at different times in the phase space. If the distance between two time points is less than a given threshold, draw a point in the RP to indicate the existence of a recursive relationship between these two time points. These points form an image on a two-dimensional plane, which intuitively displays the repetitive patterns and structures in the time series. Assuming a time series $\left\{x_{t}|t=1,2,3\cdots ,n\right\}$, construct its phase space as ${\left\{X_{i}\right\}}_{i=1}^{N}$. If the distance between any two phase points $X_{i}$ and $X_{j}$ is less than the given threshold ε, then the two points are said to be recursive. According to the Heaviside function, their values at position (i,j) are 1, and the image is a black dot. Conversely, if they are 0, they appear as a white dot. The process is as follows:

The algorithm for reconstructing the phase space of a one-dimensional time series x is:

(1) $$X_{i}=\left\{x_{i},x_{i+\tau },x_{i+2\tau }\cdots ,x_{i+\left(m-1\right)\tau }\right\}$$

In the formula, $X_{i}$ is the reconstructed point; m represents the embedding dimension; and τ is the phase space delay. After completing the phase space construction, calculate Heaviside using the Heaviside function:

(2) $$R_{i,j}=\Theta \left(\left\|X_{i}-X_{j}\right\|-\varepsilon \right),i,j=1,2,\cdots ,N$$

In the formula, $R_{i,j}$ is the positional element of an N * N-order matrix, ‖·‖ is a 2−norm calculation, and Θ is the Heaviside function, for which the value is 0 or 1. The Heaviside function is:

(3) $$Heaviside=\cdots \{\begin{array}{c} 1\;x\geq 0\\ 0\;x<0 \end{array}$$

When $R_{i,j}=1$, it indicates that the distance norm between two points is less than the threshold ε, and there is a recursive relationship between the two points; when $R_{i,j}=0$, it indicates that there is no recursive relationship between the two points.

Based on the principle of RPs, this article makes the following modifications to construct a Threshold-Free recursive plot: first, normalize the vibration signal data, and then reconstruct it into the phase space $X_{t}$:

(4) $$X_{t}=\{x_{t},x_{t+1}|t=1,2,3\cdots n-1\}$$

In the equation, X represents the reconstructed phase space, and according to Takens’ embedding theorem, for low-dimensional dynamical systems such as vibration signals, m=2 is sufficient to expand the core topology of the attractor and meet the requirement of a Threshold-Free Recursive Distance Matrix for expressing state differences; however, vibration signals generally have high sampling rates, and the interval between adjacent sampling points is small enough. Therefore, taking τ=1 can not only preserve the dynamic continuity of state evolution, but also avoid the loss of correlation caused by large delays. At the same time, low-dimensional embedding simplifies the calculation and does not lose key structural information.

Construct a Threshold-Free Recurrence Distance according to Formula (5):

(5) $$D_{i,j}={\left\|X_{i}-X_{j}\right\|}^{2}$$

In the formula, ‖·‖ represents the Euclidean norm. Unlike RPs, this method does not perform binarization of the Heaviside step function, but preserves the continuous numerical features of distance. In order to eliminate the influence of dimensionality and enhance the comparability of visualization, the distance matrix is normalized:

(6) $${\tilde{D}}_{i,j}=\frac{D_{ij}-D_{min}}{D_{max}-D_{min}}$$

Finally, D is presented in the form of a heatmap, where the depth of colors intuitively reflects the degree of similarity between states at different times: the darker the color, the closer the states at two times are; on the contrary, it indicates a significant difference in status. Through this method, weak nonlinear dynamic changes and transient characteristics in vibration signals can be more finely captured. More precisely, the method proposed in this article is an extension of recursive analysis in terms of functionality, aiming to provide richer information than a binary RP. This method can be used to explore the intrinsic structure of data, especially in situations where thresholds are difficult to confirm.

The basic principle of CNN image recognition is to simulate the human visual system, using local perception, parameter sharing, and hierarchical feature extraction mechanisms to automatically learn and recognize semantically meaningful patterns from images. It does not rely on manually designed features, but gradually transforms the original pixels into advanced semantic representations through a multi-layer structure.

**Figure 1:**
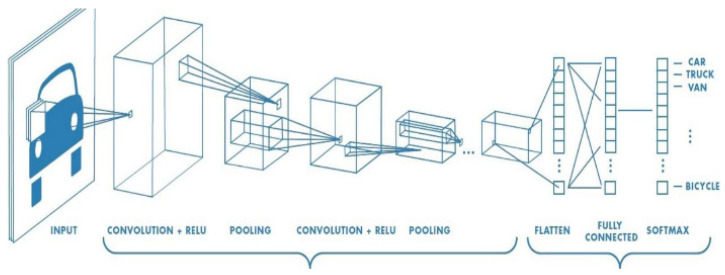
Basic Principles of CNN Image Recognition.

Initially, there is the convolutional layer, which utilizes multiple learnable convolution kernels to slide across the image, executing local weighted summation operations to generate feature maps. Each convolution kernel is dedicated to detecting specific visual patterns, such as horizontal/vertical edges, color variations, or textures. For instance, a 2 × 2 convolution kernel slides across the image with a stride of 1, progressively computing the dot product while preserving spatial positional information. Following the convolutional layer, non-linear complex functions capable of fitting the network are introduced, commonly referred to as ReLU. ReLU retains positive signals while suppressing negative ones, thereby enhancing the model’s expressive capacity and mitigating the issue of gradient vanishing. Finally, the pooling layer performs downsampling on the feature maps to reduce data dimensionality, decrease computational load, and enhance translation invariance. Common pooling techniques encompass max pooling and average pooling. These three layers constitute a fundamental module, and the stacking of multiple such modules forms a deep network, as illustrated in Figure 1.

**Figure 2:**
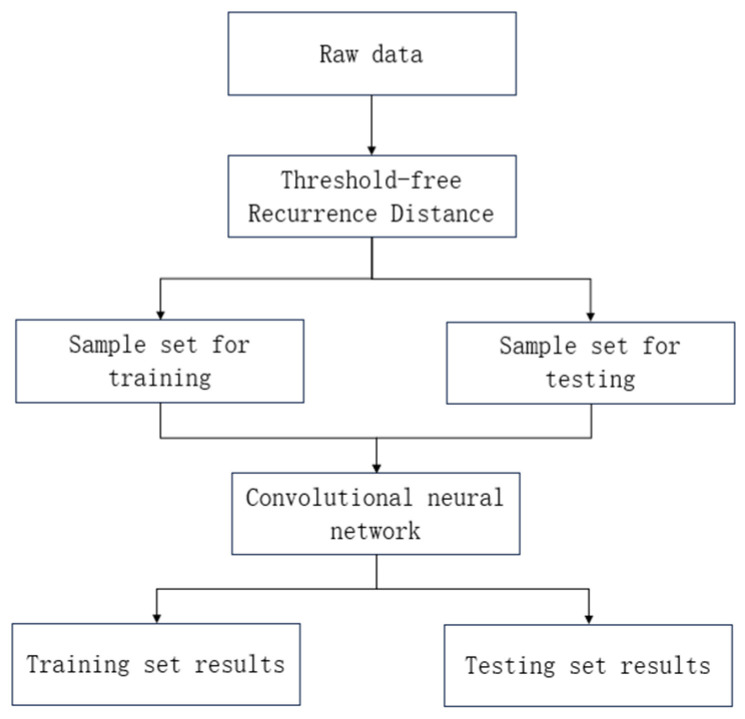
Model Algorithm Process.

A TFRD-CNN fault diagnosis model was constructed by combining Threshold-Free Recurrence Distance with convolutional neural networks, as shown in Figure 2.

The diagnostic model of TFRD-CNN proposed in this article first converts the collected vibration signals under fault conditions into TFRD. Then, the obtained images are divided into training and testing sets, and the CNN model is trained using the training set data. Finally, the diagnostic accuracy of the model is analyzed using the testing set. The specific steps are:

1. Using the sliding window method to segment the signal into segments of a certain length, forming the original vibration signal sample set.
2. Obtain a Threshold-Free Recurrence Distance for each set of raw vibration signals obtained using the method described earlier.
3. Divide the obtained Threshold-Free Recurrence Distance into a training set and a testing set according to a certain proportion.
4. Initialize the internal parameters of the CNN model.
5. Using CNN to train on the training set data, the CNN parameters are continuously updated and adjusted as the training progresses. After completion, the test set is input to verify the accuracy of the model training, achieving the required preservation of the model.

## 3. Case Analysis

To verify the generalization ability and robustness of the model under different data distributions, this section conducted tests on the publicly available bearing dataset of Case Western Reserve University and the self-built planetary gearbox fault simulation test bench dataset. There are significant differences between the two in terms of device type, faulty components, sensor characteristics, and collection environment, covering different noise bases and operating conditions. The model achieved stable classification performance on both datasets, indicating its strong generalization ability for data across devices/operating conditions.

### 3.1. Validation of the Bearing Dataset at Western Reserve University

**Figure 3:**
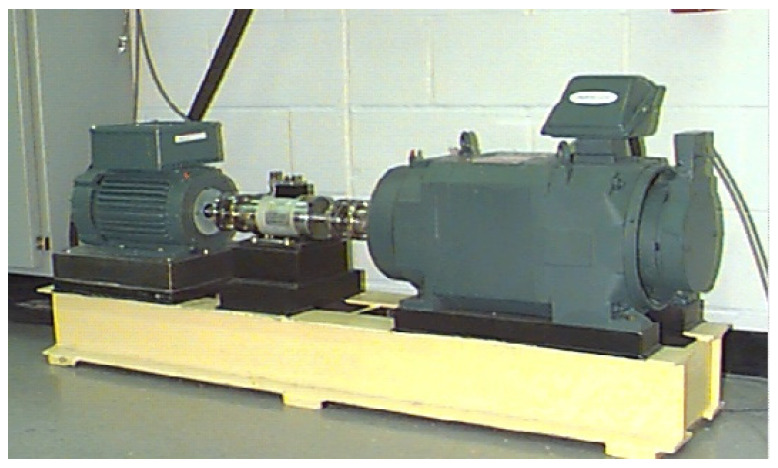
Bearing vibration test bench.

The bearing vibration dataset from Western Reserve University was employed to simulate faults through electric discharge machining. Specifically, pits with diameters of 7, 14, 21, 28, and 40 mil (where 1 mil equals 0. 0254 mm) were machined into the inner race, rolling elements, and outer race of the bearings, respectively. Vibration measurements were taken at three distinct locations: the drive end, fan end, and base. The faulty bearing was then reinstalled on the test motor, and vibration data was recorded across a load range of 0 to 3 horsepower and motor speeds between 1720 and 1797 RPM. The experimental setup shown in Figure 3, consists of a 2-horsepower motor (located on the left), a torque sensor, a load cell (located on the right), and a control circuit. The dataset can be accessed in the Supplementary Materials.

**Figure 4:**
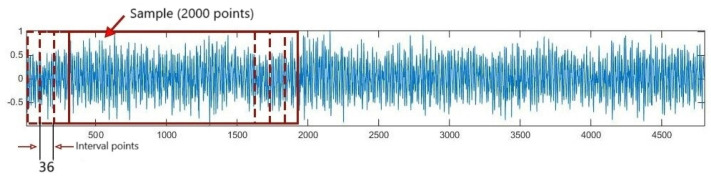
Schematic diagram of sample length extraction.

**Figure 5:**
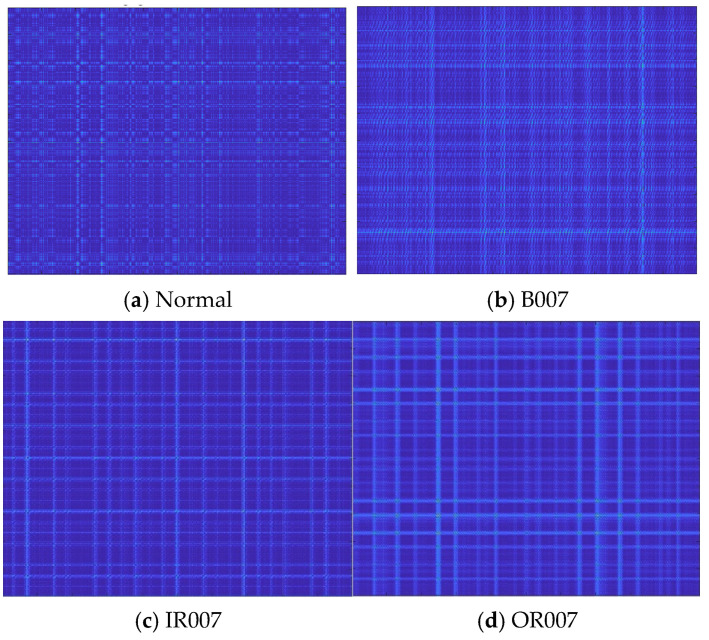
The Threshold-Free Recurrence Distance for four states: (**a**) Threshold-Free Recurrence Distance in normal state; (**b**) Threshold-Free Recurrence Distance in B007 state; (**c**) Threshold-Free Recurrence Distance in IR007 state; and (**d**) Threshold-Free Recurrence Distance in OR007 state.

This study investigates four distinct bearing conditions at the driving end measurement point, including the Normal state, a rolling element with a 0.007 mil groove (B007), an inner ring with a 0.007 mil groove (IR007), and an outer ring with a 0.007 mil groove (OR007). Divide the data of each state into samples, and collect 2000 points for each sample (each state data consist of a total of 500 samples) using the sliding window method for oversampling to increase the number of samples that can be obtained, as shown in Figure 4. The details of the sample settings are shown in Table 1. After obtaining the samples, Formulas (4) and (5) were used to generate corresponding Threshold-Free Recurrence Distances for each state. Figure 5 illustrates the Threshold-Free Recurrence Distance for all four states. Then, we divided the samples into a training set (0.65) and a testing set (0.35). In order to avoid the problem of window overlap caused by sliding window sampling, which leads to information leakage between the training set and the testing set, after generating the samples, we used the first 65% of the image data of each class of samples as the training set and the remaining as the testing set.

From the figure, it can be seen that the main diagonal of the heatmap is always at its minimum value (normalized to 0), representing a state that completely coincides or is consistent with itself; this method differs from traditional RPs with only black and white colors, and the gradient of colors in the image reveals the degree of closeness of the state. In summary, this method can distinguish between “very similar” and “relatively similar” states without setting a threshold, which is crucial for fault detection of vibration signals.

The proposed CNN architecture comprises four convolutional pooling layers and two fully connected layers. The network parameters include a learning rate of 0.0001, the rectified linear unit (ReLU) activation function, a dropout mechanism to prevent overfitting, and a softmax function for generating classification outputs. Training was conducted over 3000 iterations, with the complete configuration details presented in Table 2.

**Figure 6:**
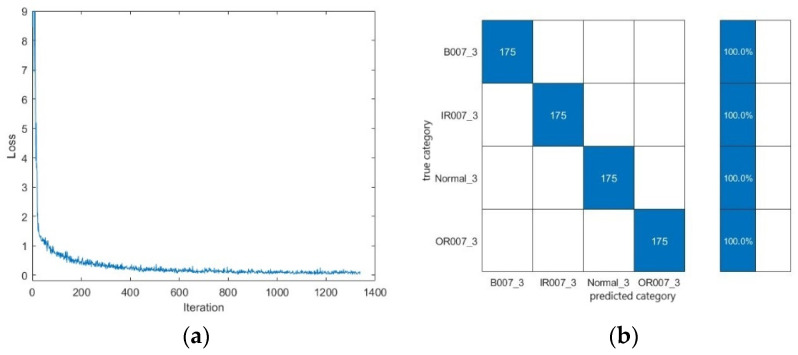
CNN loss curve and confusion matrix: (**a**) loss curve; (**b**) confusion matrix.

Upon initiating the training process, the loss curve throughout the training phase exhibits the trend illustrated in Figure 6a, while the confusion matrix generated from the test set is presented in Figure 6b.

As demonstrated in Figure 6a, at the 600th iteration, the loss curve of the model reached zero, indicating that the classification accuracy of the model has reached 100%. Upon examining the confusion matrix presented in Figure 6b, it is evident that the fault classification accuracy has achieved a perfect score of 100%, thereby validating the efficacy of our proposed methodology on this particular dataset.

### 3.2. Verification of Transmission Fault Simulation Test Platform Data

**Figure 7:**
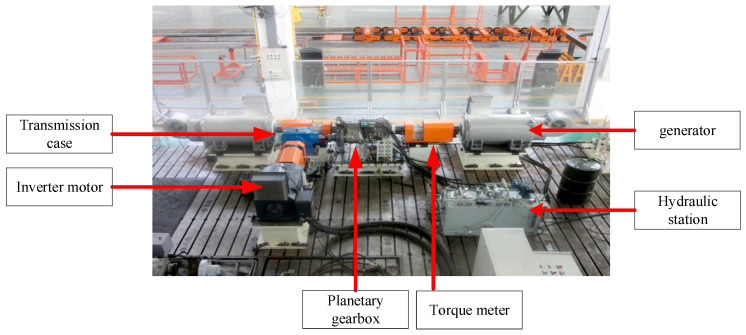
Planetary gearbox fault simulation test bench.

**Figure 8:**
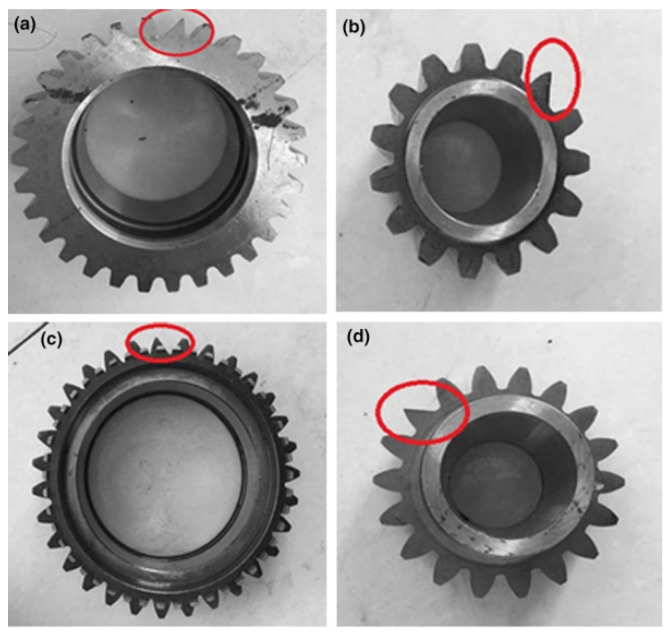
Planetary gearbox fault-type setting (teeth broken): (**a**) sun gear Z = 30, (**b**) planetary gear Z = 15, (**c**) sun gear Z = 31, and (**d**) planetary gear Z = 18.

Because the test environment of the test-bed constructed by this open dataset is relatively “clean”, that is, it has less interference, in order to verify the Threshold-Free Recurrence Distance proposed in this paper, the data of the planetary gearbox fault simulation test-bed developed by Ding Chuang et al. [25] was employed for verification purposes. The test-bed is constructed based on the gearbox of a certain type of real equipment, and its working state is consistent with the real working state, so the dataset contains noise interference caused by component friction or impact. This dataset is used to further verify the effectiveness of the proposed method for noisy data. The simulation test bench is shown in Figure 7. The dataset contains four types of fault states: Z15, Z18, Z30, and Z31 gear peeling, as shown in Figure 8; the sampling frequency is 20 kHz.

The red circles marked in Figure 8 represent the tooth breakage positions of each gear. We will take the data from a measurement under the conditions of 4th gear, 1200 r/min speed, and 900 N·m load as an example. The sliding window sampling technique is identical to the previously employed one. Each sample length is set to 2000 points to generate Threshold-Free Recurrence Distance, with 500 samples for each type, as shown in Table 3. The CNN model and parameter settings are consistent with the previous text.

After generating 2000 samples in four different states, the sample set was divided into a training set of 0.65 and testing set of 0.35.

**Figure 9:**
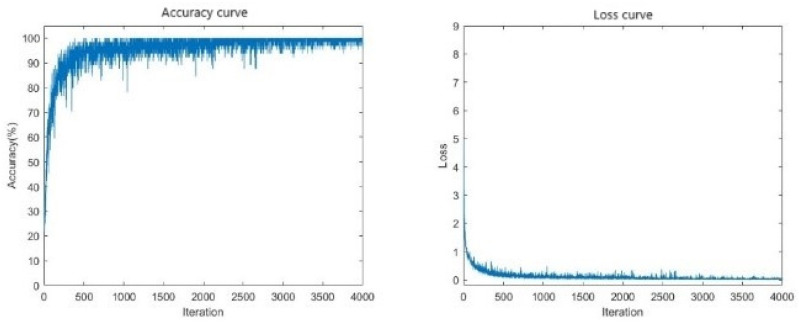
Model training accuracy and convergence curve.

Using the same CNN structure as before, after establishing the CNN architecture and its related parameters, the training process was initiated, and the convergence curve and corresponding training accuracy were obtained, as shown in Figure 9.

**Figure 10:**
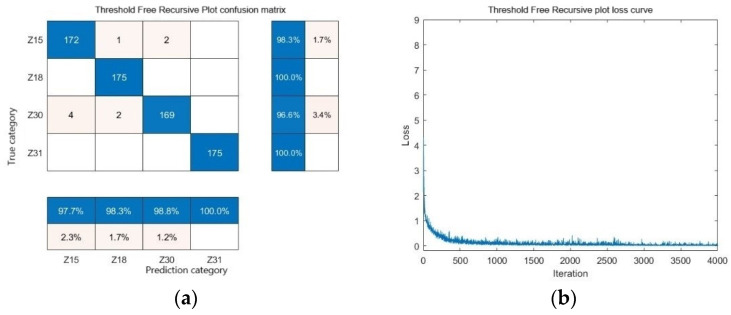
Loss curve and confusion matrix: (**a**) confusion matrix; (**b**) loss curve.

Using the constructed Threshold-Free Recurrence Distance sample test set to test the CNN model trained earlier, the confusion matrix obtained is shown in Figure 10.

As illustrated in Figure 10, the diagnostic recall rate for transmission fault data achieves 98.73%, with a corresponding fault accuracy rate of 98.7%.

**Figure 11:**
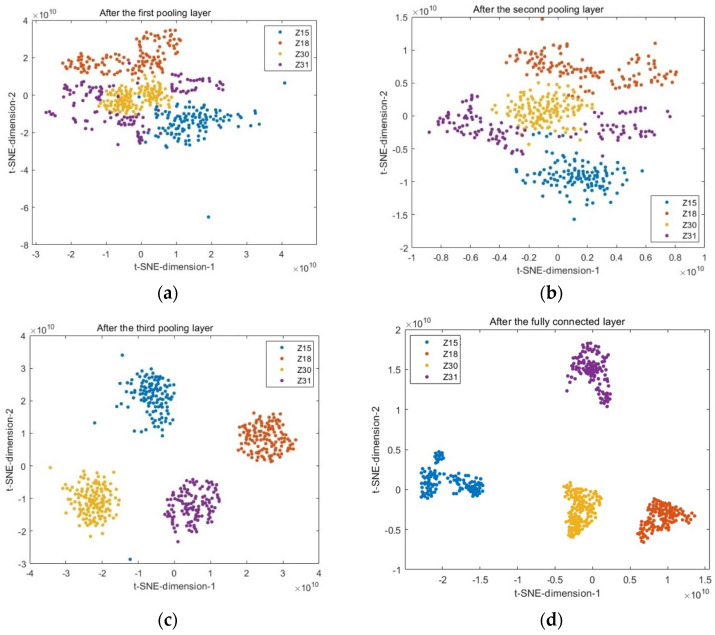
T-SNE feature visualization of four state classification processes: (**a**) Visualization results of t-sne after the first pooling layer; (**b**) visualization results of t-sne after the second pooling layer; (**c**) visualization results of t-sne after the third pooling layer; and (**d**) visualization results of t-sne after the fully connected layer.

In order to reflect the learning process of CNN on different data features, T-SNE nonlinear dimensionality reduction visualization technology is used to display the structural characteristics of high-dimensional data in the testing stage of the model, which can more intuitively observe the sample distribution and category association, as shown in Figure 11.

As demonstrated in Figure 11, the progression of data through the network architecture, from the initial convolutional pooling layer to the final fully connected output layer, reveals increasingly distinct distribution boundaries among different states, ultimately enabling effective separation of the four distinct data states.

In order to verify the reliability of the model in this paper under different working conditions, the measured data under the conditions of 4 gears, a rotating speed of 1800 r/min and 900 N·m load are analyzed. The sample structure is consistent with the previous text. A total of five states of sample data are constructed, and the data of the normal state are added, which are analyzed together with the other four states. We divide the samples into training and testing sets in a ratio of 0.8. The sample set construction is shown in Table 4.

**Figure 12:**
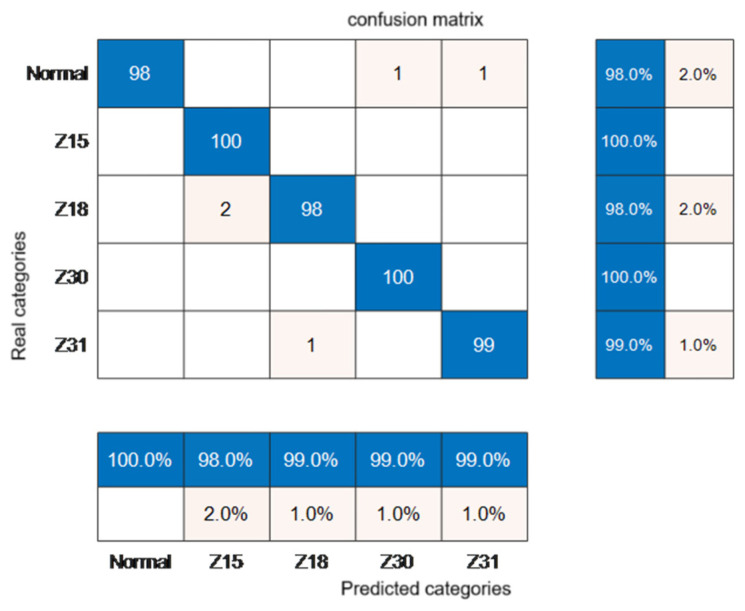
Classification confusion matrix of five states.

Input the divided sample set into the convolutional neural network model with set parameters, and finally get the classification confusion matrix of the five states of the transmission test bench, as shown in Figure 12.

**Figure 13:**
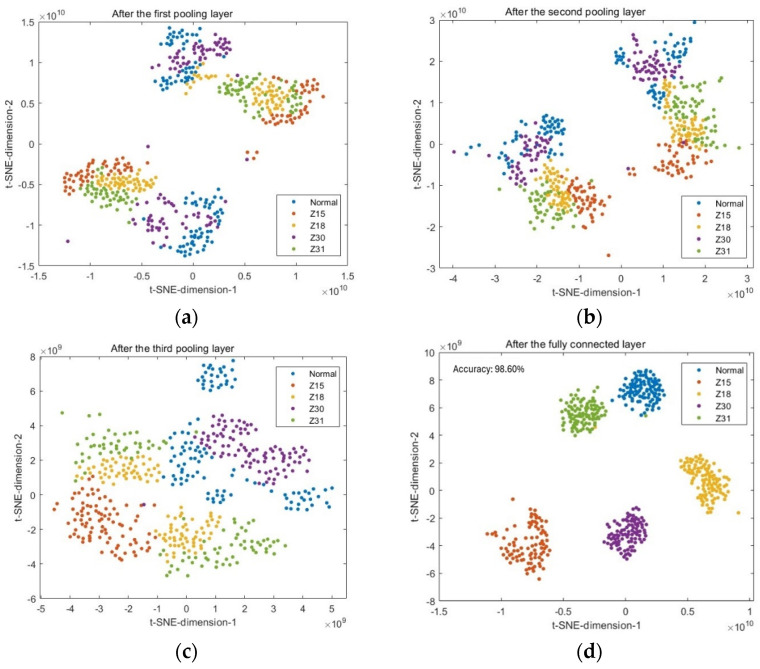
T-SNE feature visualization of five state classification processes: (**a**) Visualization results of t-sne after the first pooling layer; (**b**) visualization results of t-sne after the second pooling layer; (**c**) visualization results of t-sne after the third pooling layer; and (**d**) visualization results of t-sne after the fully connected layer.

It can be seen from the figure that the recognition accuracy and recall rate of the model for each state are above 98%. Similarly, the convolutional neural network model in the final test process is visualized with T-SNE features layer by layer, and the results are shown in Figure 13.

As demonstrated in Figure 13, the progression of data through the network architecture, from the initial convolutional pooling layer to the final fully connected output layer, reveals increasingly distinct distribution boundaries among different states, ultimately enabling effective separation of the five distinct data states.

Through the above comparison and verification, it shows that the Threshold-Free Recurrence Distance proposed in this paper has strong reliability and robustness, and shows excellent diagnostic performance in the data under different working conditions of the test bench.

### 3.3. Comparative Verification of Different Coding Methods

**Figure 14:**
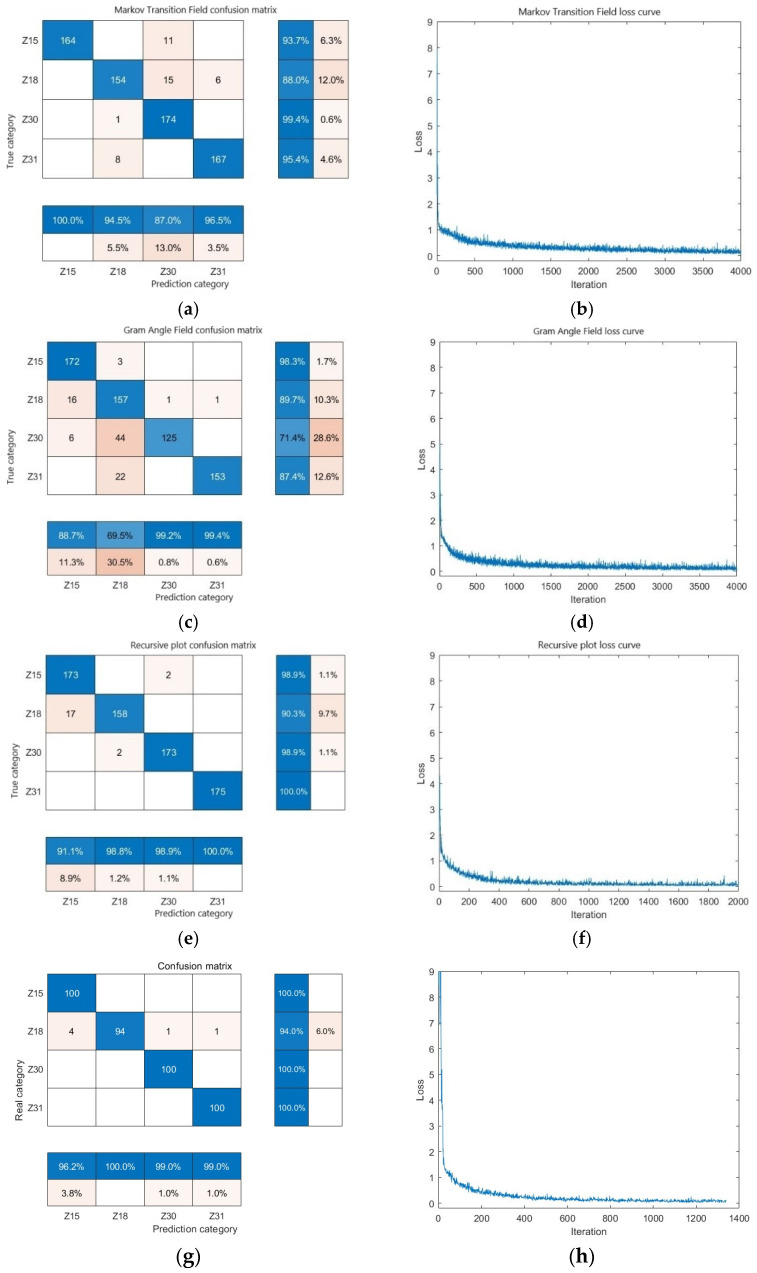
The results of the other three encoding methods: (**a**) MTF-CNN confusion matrix; (**b**) MTF-CNN test loss curve; (**c**) GAF-CNN confusion matrix; (**d**) GAF-CNN test loss curve; (**e**) RP-CNN confusion matrix; (**f**) RP-CNN test loss curve; (**g**) URP-CNN confusion matrix; and (**h**) URP-CNN test loss curve.

To validate the efficacy of the enhanced methodology introduced in this study, we implemented a comparative analysis using a CNN model trained with four image encoding techniques: Markov transition field, Gram angle field, recursive plot and URP. The recursive plot threshold was determined based on the mean value of time-domain vibration data, and the resulting samples were subsequently evaluated using a test dataset. The performance metrics, including confusion matrix results and loss curve analysis, are presented in Figure 14.

Upon analyzing the four confusion matrices discussed earlier, it is evident that the diagnostic accuracy for the Z15 gear peeling state, when employing the Threshold-Free Recurrence Distance, exhibits a slight improvement compared to the recursive graph-based fault diagnosis. However, the diagnostic performance for other state categories remains largely comparable. To enhance the reliability of the findings and thoroughly validate the proposed model’s advantages, the four models underwent 50 independent training and testing iterations. This process yielded the corresponding recall and accuracy metrics for each of the 50 fault diagnosis outcomes. After computing the average, Table 5 presents the final recall and accuracy values for the fault diagnosis of five models, and calculates the average F1 score for each model by computing the F1 score for each category.

Table 5 shows that the TFRD-CNN model achieved the highest diagnostic performance, and through F1 score comparison, the model proposed in this paper has superior performance compared to other methods.

## 4. Discussion

In order to overcome the subjectivity of threshold ε selection and the resulting binary information loss in traditional Recursive Plots (RPs), this paper adopts a Threshold-Free Recursive Distance Visualization method based on the basic principles of recursive graphs. The experimental results demonstrate the robustness and generality of the improved method proposed in this paper, but at the same time, it also has certain limitations:

1. By preserving the continuous numerical characteristics of distance without manual threshold selection or binary processing of Heaviside step function, a heat map is drawn to display the recursive distance matrix. The color intensity represents the similarity of the vibration system between different time points. The darker the color, the closer the states at two time points. Otherwise, it indicates a large difference in states. This method can more finely capture the weak nonlinear dynamic changes and transient features in vibration signals. Therefore, compared with Gram angle fields, Markov transition fields, recursive graphs, and current threshold-free recursive graphs, it has better fault identification ability.
2. This article conducted tests on both the self-built planetary gearbox fault simulation test bench dataset and the publicly available bearing dataset from Case Western Reserve University. There are significant differences between the two in terms of device type, faulty components, sensor characteristics, and collection environment, covering different noise bases and operating conditions. The proposed model achieved stable classification performance on both datasets, indicating strong robustness and generalization.
3. The method proposed in this article preserves continuous distance values, resulting in a continuous gradient of color in the drawn heatmap, with low visual contrast. At the same time, using the heatmap as input for the CNN increases the computational difficulty and GPU memory pressure during training.

## Figures and Tables

**Table 1:** Sample settings in four states.

Bearing Fault Data Sample Setting				
	Normal	B007	IR007	OR007
Sample points	2000	2000	2000	2000
Number of samples	500	500	500	500

**Table 2:** CNN model settings.

Serial Number	Hierarchy Type	Key Parameters/Operations	Output Size Change	Effect
1	Input	224 × 224 × 3	224 × 224 × 3	Data entrance
2	Conv Block 1	Conv Kernel 5 × 5, 32 filters, Padding ’same’ Pool: Window 2 × 2 Stride 2	112 × 112 × 3	Extract low-level features and reduce dimensionality
3	Conv Block 2	Conv Kernel 3 × 3, 64 filters, Padding ’same’ Pool: Window 2 × 2 Stride 2	56 × 56 × 64	Extract intermediate features
4	Conv Block 3	Conv Kernel 3 × 3, 128 filters, Padding ’same’ Pool: Window 2 × 2 Stride 2	28 × 28 × 128	Extract advanced features
5	Conv Block 4	Conv Kernel 3 × 3, 256 filters, Padding ’same’ Pool: Window 2 × 2 Stride 2	14 × 14 × 256	Extract semantic features
6	FC Layer	512 neurons + Dropout Rate: 0.5	1 × 1 × 512	Global feature integration and prevention of overfitting
7	Output	numClasses neurons + Softmax	1 × 1 × NumClasses	Output classification probability

**Table 3:** Four types of fault samples.

Gear Spalling Fault Data Sample Setting				
	Z15	Z18	Z30	Z31
Sample points	2000	2000	2000	2000
Number of samples	500	500	500	500

**Table 4:** Five types of fault samples.

Gear Spalling Fault Data Sample Setting					
	Normal	Z15	Z18	Z30	Z31
Sample points	2000	2000	2000	2000	2000
Number of samples	500	500	500	500	500

**Table 5:** Comparison of five image encoding methods.

Fault Category	MTF-CNN		GADF-CNN		RP-CNN		URP-CNN		TFRD-CNN	
	Accuracy Rate	Recall Rate	Accuracy Rate	Recall Rate	Accuracy Rate	Recall Rate	Accuracy Rate	Recall Rate	Accuracy Rate	Recall Rate
Z15	95.0%	93.7%	87.7%	96.3%	96.1%	97.9%	97.5%	98.5%	97.7%	98.5%
Z18	94.5%	88.0%	70.5%	91.7%	98.1%	90.3%	98.6%	99.2%	98.9%	100.0%
Z30	87.0%	99.4%	96.2%	73.4%	98.5%	98.9%	98.8%	98.8%	98.8%	98.6%
Z31	96.5%	95.4%	94.4%	87.4%	99.1%	99.3%	99.1%	99.4%	100.0%	100.0%
F1-score	0.9355		0.8639		0.9723		0.9874		0.9906	

## Data Availability

The data that support the findings of this study are available from the corresponding author upon reasonable request.

## Supplementary Materials

The following supporting information can be downloaded at: https://gitcode.com/open-source-toolkit/a8c15 (accessed on 8 June 2026).

## Abbreviations

The following abbreviations are used in this manuscript:

CNN	Convolutional Neural Network
RPs	Recursive Plots
TFRD	Threshold-Free Recurrence Distance
MTF	Markov Transition Field
GAF	Gram Angle Field
